# Ethical preparedness in health research and care: the role of behavioural approaches

**DOI:** 10.1186/s12910-022-00853-1

**Published:** 2022-11-17

**Authors:** G. Samuel, L. M. Ballard, H. Carley, A. M. Lucassen

**Affiliations:** 1 Clinical Ethics, Law and Society (CELS), Wellcome Trust Centre for Human Genetics, University of Oxford, Oxford, OX3 7BN UK; 2 Clinical Ethics, Law and Society (CELS), Primary Care, Population Sciences and Medical Education, Room AB 209, MP 801, Faculty of Medicine, University of Southampton, Southampton General Hospital, South Academic Block, Tremona Road, Southampton, SO16 6YD UK; 3 NIHR Southampton Biomedical Research Centre, University of Southampton and University Hospital Southampton NHS Foundation Trust, Southampton, UK; 4 Department of Global Health and Social Medicine, King’s College London, London, UK

**Keywords:** Ethical preparedness, COVID-app, Genomics, Behavioural science, Implementation science, COM-B model, Health psychology

## Abstract

**Background:**

Public health scholars have long called for preparedness to help better negotiate ethical issues that emerge during public health emergencies. In this paper we argue that the concept of ethical preparedness has much to offer other areas of health beyond pandemic emergencies, particularly in areas where rapid technological developments have the potential to transform aspects of health research and care, as well as the relationship between them. We do this by viewing the ethical decision-making process as a behaviour, and conceptualising ethical preparedness as providing a health research/care setting that can facilitate the promotion of this behaviour. We draw on an implementation science and behaviour change model, COM-B, to demonstrate that to be ethically prepared requires having the *capability* (ability), *opportunity*, and *motivation* (willingness) to work in an ethically prepared way.

**Methods:**

We use two case examples from our empirical research—one pandemic and one non-pandemic related—to illustrate how our conceptualisation of ethical preparedness can be applied in practice. The first case study was of the UK NHSX COVID-19 contact tracing application case study involved eight in-depth interviews with people involved with the development/governance of this application. The second case involved a complex case regarding familial communication discussed at the UK Genethics Forum. We used deductive qualitative analysis based on the COM-B model categories to analyse the transcripbed data from each case study.

**Results:**

Our analysis highlighted that being ethically prepared needs to go beyond merely equipping health professionals with skills and knowledge, or providing research governance actors with ethical principles and/or frameworks. To allow or support these different actors to utilise their skills and knowledge (or principles and frameworks), a focus on the physical and social opportunity is important, as is a better understanding the role of motivation.

**Conclusions:**

To understand ethical preparedness, we need to view the process of ethical decision-making as a behaviour. We have provided insight into the specific factors that are needed to promote this behaviour—using examples from both in the pandemic context as well as in areas of health research and medicine where there have been rapid technological developments. This offers a useful starting point for further conceptual work around the notion of being ethically prepared.

## Background

Public health scholars have long called for preparedness to better help negotiate ethical issues that emerge during public health emergencies [1–6]. Being ethically prepared for a public health emergency is viewed by some to be just as important as being prepared to acquire resources, such as personal protective equipment [7]. Scholars have argued that by applying various values and principles, such as openness, transparency, and accountability, ethical issues can be anticipated, identified, and addressed in public health emergency decision-making [1, 4]. Furthermore, by providing the moral language to describe and resolve situations in which values conflict during an emergency, this can provide ethical legitimacy to the policy decision-making processes [2, 8]. A lack of such preparedness has been perceived to lead to low levels of trust and morale in pandemic situations, as well as fear and misinformation [6]. It may also leave the public vulnerable to unequitable and ‘regrettable’ decision-making by governments and health providers [9], especially when rapidly changing situations make routinised decision-making difficult [10].

We argue that the concept of ethical preparedness has much to offer other areas of health research and medicine in addition to public health emergencies, particularly where rapid technological developments have the potential to transform practices. This is because health researchers and healthcare professionals need to be prepared, i.e., expectant and ready, to face new challenges born of the complexity, uncertainty and longevity of technologies and their implementation into health contexts, and it is vital that they do so in ethically appropriate ways.

In this paper we problematise the concept of ethical preparedness in the literature to date, which mainly constructs the concept as a need to develop frameworks and principles [2, 8, 11–16]—exceptions include Leach et al. [17] and Coggon et al. (2017), or refers to it as ‘legalistic’ [18], or ‘bureaucratic’ processes. Such processes are insufficient as a proxy for ethical preparedness and do not ensure the implementation of ethical decision-making in practice [11, 13, 14, 17, 19]. This is because ethics is also about the ethical questions that are faced by *individuals* in their *day-to-day practices* [18]. Ethics is situated and contextual: it might draw upon laws and guidance but will likely require knowledge of particular factors in a specific situation (situated knowledge) to be enacted, and which in turn can sometimes reveal the deficits of a framework or policy.

We experiment with the concept of ethical preparedness by shifting it away from one that involves the development of principles and frameworks. Rather, following Leach et al. [17], we view the process of ethical decision-making as something that is enacted by a person, group or organisation, i.e., *a behaviour*. Being ethically prepared therefore means *establishing settings* that make it more likely for a person, group, or organisation to adopt ethical decision-making behaviour. Importantly, ethical preparedness is not about the outcome—and says nothing about how such moral decisions can or should be made, nor on the basis of what values and/or principles—but rather it allows us to consider how we can promote factors which support the ethical decision-making process in a particular setting.

To do this, we turn to behaviour change and implementation models. Such models are designed to identify what attributes are required for the implementation of certain behaviours into practice. Behaviour change can be targeted at individuals, groups, organisations, and at a societal level, but it will always be individuals doing the behaviour, even when targeting an organisation. We use these models to understand what attributes affect the setting in which the behaviour of the ethical decision-making process is occurring, and then include these attributes in our conceptualisation of ethical preparedness. Specifically, we draw on the COM-B model because it has been systematically developed as an overarching framework (Theoretical Domains Framework (TDF)) combining 33 theories to conceptualise factors affecting implementation in practice. The COM-B model is widely accepted in the field of behaviour change [20].

Next, we apply the COM-B model to two illustrative case studies: if we view ethical preparedness as being a setting which promotes a person, group or organisation’s ethical decision-making behaviour, then to understand particular attributes within particular settings requires in-depth empirical analysis of contextualised cases. This can provide answers to the question ‘what could have been done better?’ in situations where ethical preparedness was lacking or limited in practice. It can also provide a forward-looking empirical model to study future cases and understand what would have been required to be ethically prepared in any specific context.

In applying a behavioural model approach, we do not aim to provide a reductive list of influential factors. We recognise that behaviour is the result of a complex interrelationship between a self and others, affected by various social, cultural, resource, political (and other) factors that interact at the micro, meso and macro level (see, for example, the ‘ethics of care’ literature). Though we emphasise that COM-B aims to encapsulate these factors (see below and Table 1) and allows us to group them with an understanding that the categories are interrelated and overlapping; and that sometimes some factors will be more prominent and important than others. These groupings make for more manageable understandings, which allows us to articulate and discuss across disciplines, and in a way that permits for a clearer understanding of ethical preparedness.

**Table 1:** Definitions of behaviour and each element of the COM-B model [22]

Capability	Physical capability	Psychological capability
An attribute of a person that together with opportunity makes a behaviour possible or facilitates it	A person’s physique and musculoskeletal functioning	Mental functioning: for example, skills, understanding, memory, knowledge and behavioural regulation

Opportunity	Physical opportunity	Social opportunity
An attribute of an environmental system that together with capability makes a behaviour possible or facilitates it e.g., social, political, cultural factors	Inanimate parts of the environmental system and time. For example, financial, material resources and environmental context	Other people and organisations. For instance, culture, social norms and social influences. e.g., ‘not my responsibility’, ‘others are not doing it, why should I?’

Motivation	Reflective motivation	Automatic motivation
An aggregate of mental processes that energise and direct behaviour	Conscious thought processes. For example, plans, evaluations, identity, beliefs in capability, goals, beliefs about consequences	Habitual, instinctive, drive-related and affective processes. For example, desires, habits and emotion regulation

Below, we first describe the COM-B model and how we have drawn on the model to conceptualise our definition of ethical preparedness, we then present our case studies.

### The COM-B Model

**Fig. 1:**
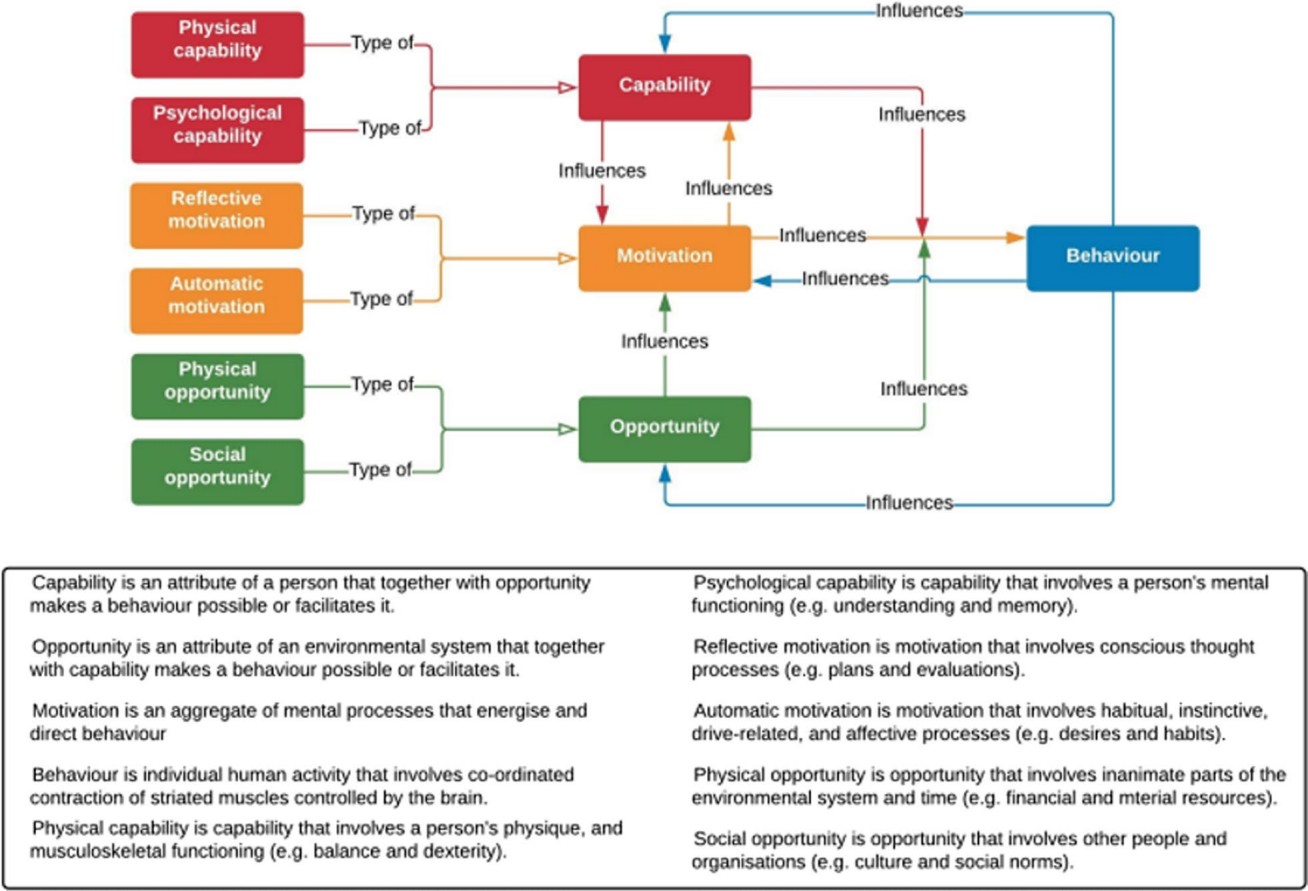
COM-B model [21]

Central to the COM-B model is that for a behaviour to happen, a person, group or organisation must have the *capability* and *opportunity* to perform a behaviour, as well as sufficient *motivation* to perform it above the motivation to perform other behaviours (Capability, Opportunity, Motivation = Behaviour model; Fig. 1) [21]. Figure 1 shows the elements of COM-B and the six sub-elements: physical capability, psychological capability, reflective motivation, automatic motivation, physical opportunity, and social opportunity.

**Fig. 2:**
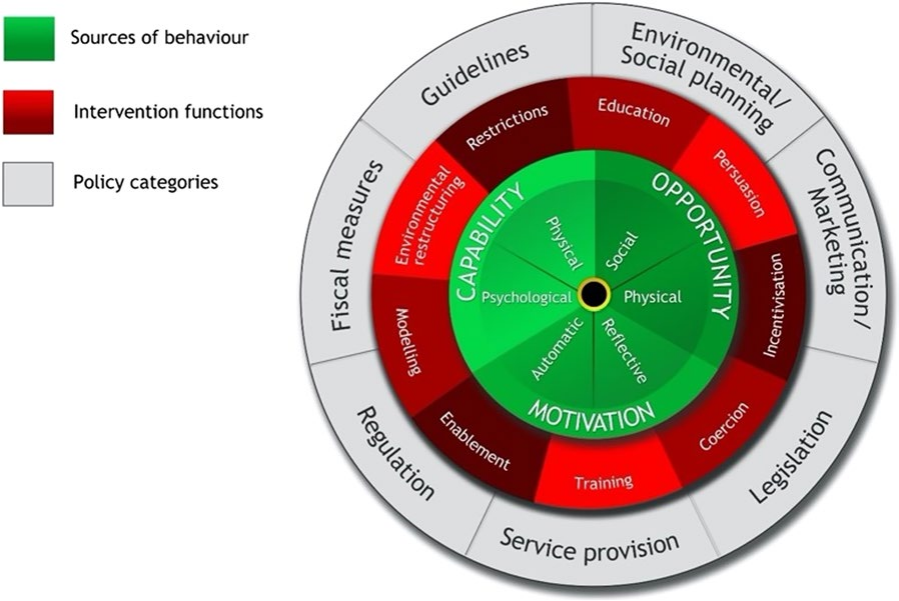
The Behaviour Change Wheel [22]

**Fig. 3:**
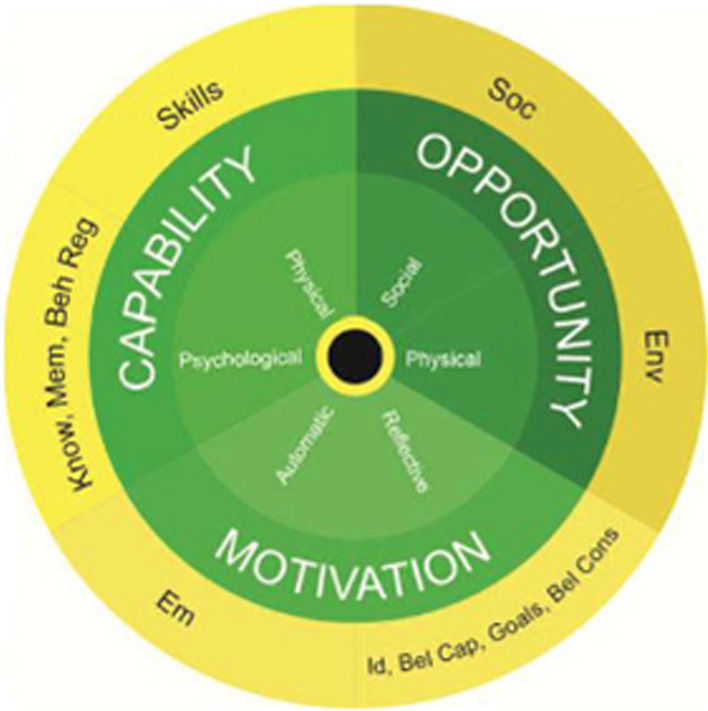
Theoretical domains framework (yellow circle) linked to COM-B (green circle) [26]

The Behaviour Change Wheel—at which COM-B is at the centre (see Fig. 2)—was developed by Michie, van Stralen and West in 2011. Each element of the COM-B model can be subdivided into theoretical domains (TDF), a more fine-grained version of the COM-B model (see Fig. 3 [23]). Specifically, *capability* encapsulates the domains of skills, knowledge, memory and behavioural regulation; *opportunity* includes social influences and environmental context, such as the wide range of social, religious, cultural, political, policy, and/or professional factors that were described above; and *motivation* includes emotion regulation, identity, beliefs in capability, goals, and beliefs about consequences (see Table 1). Together, these three domains cover a range of individualistic, social, and political factors functioning at the micro, meso, and macro levels. As Fig. 1 shows, both capability and opportunity can influence motivation, and motivation can influence capability. This means that the influences on a behaviour from the social domain can govern which competing motivations drive the performance of a behaviour (or not). The COM-B/TDF model has been used to explore issues similar to the cases we discuss below, such as the barriers and facilitators to mainstreaming genetics and genomics [24] and implementing crisis standards of care during the COVID-19 pandemic in acute care hospitals [25].

The COM-B model facilitates the understanding of a behaviour in context, which then allows for intervention. COM-B is at the centre of the wheel in green, with intervention functions in red and policy categories in grey. Each layer links to the next, for example, psychological capability can be achieved through education, training, and enablement interventions. Policy categories and intervention functions also link, meaning education can be achieved through policies regarding communication/marketing, guidelines, regulation, legislation, and service provision.

### Ethical preparedness

Applying COM-B/TDF to our articulation of ethical preparedness, an individual—including individuals who are part of groups, or organisations by way of an organisational culture—needs to have the capability, opportunity and motivation to be ethically prepared. We define ethical preparedness as having the *capability* (ability), *opportunity*, and *motivation* (willingness) to anticipate and identify ethical issues and to be able to implement ethical decision-making in practice. *Capability* refers to *how* individuals can be ethically prepared in health research or healthcare. This could be in the form of guidelines but also refers to the skills to be ethically aware, sensitive, and reflective. This might be realised through training courses, multidisciplinary meetings, or through spontaneous ethical thinking for which individuals have capability. *Opportunity* refers to the social, political, cultural, and organisational factors within health research and/or care settings that allow ethical preparedness in practice. Finally, even with ability and opportunity, ethical preparedness could be absent if there is a lack of *motivation*. For example, a healthcare professional or researcher may choose not to follow-up a potential ethical issue that arises during practice, rationalising that it falls outside of their responsibility and that to do so would require [too much] time and effort on their part.

In the following, we apply our conceptualisation of ethical preparedness to two case studies from our own empirical research.

## Case studies

We have selected our two cases to include a pandemic and non-pandemic context, as well as a research/care and individual/organisation context.

The first case study is situated in the *pandemic* context and is related to health *research*, with ethical preparedness being considered at the *organisational* level. It explores the development and governance of the original 2020 UK NHSX COVID-19 contact tracing application (app). We analyse the case through our conceptualisation of ethical preparedness, bringing clarity and understanding as to why, even when governance steps were taken to be ethically prepared (by means of the establishment of an Ethics Advisory Board (EAB) to assist with addressing ethical questions associated with the development of the app), ethical preparedness was not always evident in decision-making. We draw on empirical research that involved eight in-depth interviews with those involved with the development and/or governance of the app, those who had a consulting role associated with the app, and/or who sat on the EAB within the app’s governance structure (June–August 2020) [27]. Ethics approval was received from King’s College London research ethics office: MRA-19/20-19251.

The second case study is a *non-pandemic, healthcare* context, exploring *individual* ethical preparedness. It relates to a singular morally complex clinical case concerning familial communication in genetic practice, which was recently discussed at the UK Genethics forum. This is a multidisciplinary forum to support ‘*professionals working in clinical genetics to become more sensitive to ethical issues that arise in their practice and develop skills in discussing and resolving them*’ [28]. Over a period of twenty years, over 1000 morally complex clinical cases have been contributed to this forum, which meets three times per year to provide ethics support to professionals in the field. The forum attendance is composed of healthcare professionals, healthcare laboratory scientists, patient representatives, academic lawyers and social scientists. Since the start of the COVID-19 pandemic, the forum meetings have occurred online and have been recorded and transcribed. Analysis of the recent transcripts revealed several cases that contained rich accounts of the ethical issues and examples of ethical decision-making in particular clinical scenarios. The Genethics Forum project was approved by the Faculty of Medicine Ethics and Research Governance Committee at the University of Southampton, UK [ERGO Number: 67992].

To apply our conceptualisation of ethical preparedness to our case studies we drew on published methodologies that apply COM-B [29, 30]. LMB has extensive experience with this methodological approach.

Applying this model is a form of deductive qualitative analysis. For each case study, the authors (first case study: GS, LMB; second case study; GS, LMB and HC) independently read the relevant case material (interview/Genethics meeting transcript(s)) and coded the data into one of six sections: ‘capability’, ‘opportunity’ and ‘motivation’, each containing two distinct components. Following independent analysis, the coding was cross-referenced, and any disagreements were discussed and resolved. Several meetings were held between all authors to discuss findings. During the coding process, close attention was paid to the factors that our interviewees/contributor perceived to influence ethical decision-making in practice. For the first case study, our interviews provided rich data to help us identify influences on behaviour and the categorisation. For the second case study, we used the transcription of the case description and ensuing discussion for our analysis. There was an element of interpretation that occurred here in both studies. To address this, we presented our findings to some of our interviewees (case study 1) and contributing healthcare professional (case study 2) so they could reflect on our interpretations. Reflections were incorporated into our analysis in an iterative way. While we recognise that this does not fully address the interpretive nature of our approach, we feel that our findings are rigorous enough to offer useful insights into how COM-B/TDF can be applied, and that our findings can be followed up in further research and empirical data collection.

## 1. NHSX COVID-19 web application

In 2020, to assist with containing the spread of the novel coronavirus, policymakers around the globe began to develop mobile contact tracing ‘apps’ to support the process of contact tracing. These apps aimed to alert individuals likely to have encountered someone reporting symptoms or testing positive for the virus, and request that they self-isolate. Between March–May 2020, NHSX, a unit of the UK National Health Service (NHS) responsible for digital innovation, was given the role of developing a UK contact tracing app. This app was trialled on the Isle of Wight in May–June 2020. The trial was halted in June 2020 and the app was remodeled by NHS Test and Trace and launched in England and Wales in September 2020 [31].

During the development of the original app, an EAB was established with the published aim to provide timely advice, guidance and recommendations on ethical issues associated with the app’s development [32]. Specifically, its purpose was to ensure that the development of the app helped to control the COVID-19 epidemic and return people to normal life rapidly whilst operating in line with ethical requirements, and in a manner that was transparent and open to public scrutiny [33, 34]. Furthermore, it was to ensure that if people did choose to use the app, they could be assured that their privacy and other interests were appropriately protected. Publicly available information about the working of the EAB is available in its final report [33], and is described in more detail below.

In our exploration of how ethics was incorporated into decision-making during governance processes associated with the app, we have argued elsewhere that the EAB fulfilled some role as an advisory board within the governance structure by promoting the importance of thinking about ethical issues for those developing and/or governing the app. However, alongside this, we also emphasise that through the EAB and more broadly, it was difficult to operationalise ethics into governance processes [27].

### Capability (ability)

The remit of the EAB was to offer advice to policymakers on the various frameworks and values instilled in the decisions associated with the app. The EAB produced several publications offering an ethical toolkit (*knowledge* and *skills*) to help those governing the development of the app think through the ethical issues associated with the app, and develop a moral language for discussion [35]. For example, a letter to the UK Secretary of State from the EAB noted and described how to apply a framework of six principles to ensure that the app was developed in an ethical way. These included: value, impact, security and privacy, accountability, transparency, and control. In this way, the involvement of ethicists within the organisational governance of the COVID-19 app, via the EAB, could be argued to represent a *capability*—at the *organisational* level—to have the skills and knowledge to incorporate ethical insight into governance processes.

### Opportunity

The EAB had the *physical opportunity* to achieve its goal to advise government on ethical considerations associated with the app. Interviewees described how they had regular online meetings to discuss and deliberate issues. One interviewee described how they had ‘*worked out that [another similar] committee…took about 3 years to have the number of meetings’* the EAB had in 3 months (interviewee 1). However, while the EAB was accommodated within a governance framework, there was a lack of procedural infrastructure at an organisational level to provide the *social opportunity* for the knowledge and advice emanating from the EAB to be imparted onto those who worked tirelessly on the app’s development. In fact, as we have argued previously, ethical deliberation was not embedded into the day-to-day decision-making of app development; ‘*if they [the EAB] could have been more involved on a day-to-day basis it would have been beneficial’* (interviewee 8). The EAB final report highlighted how the Board was never provided with the level of detail they asked for concerning the technical data from the tests and trial that they would expect as an internal board who can review confidential material [33] (this could also be seen as the EAB lacking the required knowledge (*psychological capability*)). This frustrated those interviewees who were members of the EAB, as did the fact that the EAB could only give advice rather than having to be listened to (*social opportunity*); ‘*the only power the advisory board members had was really to quit as far as I see it. They could give all the best advice they wanted and that may or may not have been listened to’* (interviewee 7).

This is not to say there was no opportunity—there were successes that members of the EAB spoke about when they managed to bring about a policy change, and in this way the EAB served a purpose. However, to really ensure a shift in behaviour, and to ensure that ethics was at the forefront of those involved in decision-making, more *social opportunity* was needed in the governance structures at the organisational level to bring the ethical knowledge of the EAB to not only those working on the app, but those making decisions about the app. Key structural issues through hierarchical governance made it difficult for the EAB’s ethical framework to be incorporated into the practices of those developing the app (*physical opportunity*). An approach that promotes horizontal layers of intervention—i.e., ‘collaboration, coordination, shared responsibility for decisions and outcomes, and a willingness to work through consensus’ [36], pg. 1—would have perhaps better empowered individuals developing the app, as well as those within the EAB, to have open deliberations within the context of specific ethical issues related to the technology [37]. However, interviewees stressed that there was little motivation (see below) on the part of the UK government to implement this (‘*they [the UK government] sort of forced people into this hierarchy*’ (interviewee 2)). Furthermore, often, there was a lack of *social opportunity* because ethical engagement was de-prioritised at an organisational level. One reason for this was perhaps because the historical/cultural issue of an entrenched lack of favourable environment and social acceptability of recognising ethics as equal knowledge and of equal importance to other forms of knowledge. As such—and indicative of contemporary debates around science and technology—ethics dialogue often featured lower in the hierarchy compared to other (more scientific) epistemologies [38]. This was illustrated by the way in which interviewees often separated the need to implement ethical decision-making into practice from the need to control the pandemic. This demonstrated the absence of acceptability (*social opportunity*) to think about ethical decision-making as intrinsic to development, rather than an add on. This is described in more detail below.

### Motivation (willingness)

The environment affects motivation, as do individual and organisational factors. The environment in this case included the first UK COVID-19 lockdown (March–May/June 2022), in which residents of the UK were instructed to remain in their homes because of concerns about the COVID-19 virus, and experts had little understanding of how the COVID-19 pandemic would proceed in the coming months and years. It also included information from the media and scientists; internal government information about the progress of the pandemic; and tensions the organisation (UK government/NHSX) faced regarding how and when the app would be ready versus how to ensure ethical decision-making occurred in practice. Interviewees explained that this involved the weighing up of *perceived* competing motives (we note that these motives may not actually be competing). This included, on the one hand, not completely operationalising ethical decision-making but potentially speeding up the development of the app (there is no evidence that ethical decision-making would or would not have slowed down development), the consequences of which could lead to a potential reduction in the spread of the virus and would allow the country to reduce lockdown restrictions (something deemed ethically appropriate). On the other hand, this could be accepting the consequences of developing the app at a slower pace, with the full operationalisation of ethical preparedness. (This dichotomisation is an oversimplification, as, for example, it could be argued that there was an ethical obligation/imperative to develop the app as quickly as possible to ensure the best opportunity to minimise harm. Nevertheless, even if such a moral imperative can be argued, this does not detract from the need to ensure the app was developed and governed in a way that was open, accountable and collaborative). Some of our interviewees suggested that in this instance, while many on the EAB and those working on the app did have the motivation to apply ethical insights and decision-making in practice, this at times did need to be weighed up against the motivation to control the pandemic. For example, one of the interviewees explained that because of the situation, ethical oversight was not always at the forefront of people’s minds (their primary motivation) as much as if, for example, a digital technology had been developed for a particular disease in ordinary circumstances, in which case ‘*we would say “right, what kind of oversight is needed for that?”’* (interviewee 5).

Finally, while nearly all interviewees provided different examples of how they were motivated by past experiences to understand the need for the incorporation of key ethical principles, for example, accountability and transparency (interviewees highlighted, for example, the care.data scandal, which was an initiative that aimed to improve the use of UK General Practitioner data for research, but received harsh public criticism and was eventually halted), they spoke about the oftentimes lack of motivation of the UK government’s political agenda to implement this in practice (*reflective motivation*). For example, talking about transparency, interviewee 3 emphasised; *‘you…did get a perception as things began to get slightly less emergency-ish, that the government was essentially just ultra-cautious about revealing anything’*. Reward has been described as a key aspect to increasing motivation [21]. While the reward for many engaged in the app’s development and governance related to ensuring an ethically responsible approach to the app’s design, for the UK government—and perhaps others involved in the design process—there was just not enough reward to be more ethically prepared. Scholars have argued that public trust/support is a key motivator for organisations [39]. Perhaps, given that enough public support was present at the time when the app was being developed [27], there was little additional motivation—and therefore reward—to *act* in an ethically appropriate way.

### Summary

As discussed previously, for a behaviour to happen, capability, opportunity and motivation need to be present (COM-B model). In this case, there was sufficient capability (i.e., the ethical toolkit and instructions on how to apply the ethical framework) but opportunity was lacking (i.e., inadequate communications between the EAB and app developers, and ethics was deprioritised). Contextual pressures to try and reduce the infection rate as quickly as possible may have also had an influence on psychological capability, meaning app development and speed were prioritised and ethical aspects (for example, openness, accountability and open collaboration and input) were less valued.

Overall, this case study has highlighted the factors that made it difficult for ethical preparedness to be implemented even with the presence of an EAB. This is important because providing the psychological capability (skills, knowledge, understanding) is perhaps the easiest aspect to achieve when considering how to be ethically prepared. It is what surrounds this capability—allowing those skills and knowledge to be enacted—that is perhaps harder to achieve, because they may go against current culture and deeply ingrained ways of thinking and working. A national emergency is not the appropriate context to interrogate and change this culture. Rather, we can learn from this case study: cultures that support ethical decision-making (i.e., ethical preparedness) need to be developed in advance of such emergencies, and practiced routinely, so that they are engrained if and when future emergencies arise.

## 2. Familial communication in genetics case study

The second case study describes a morally complex clinical case encountered by a Clinical Genetics healthcare professional and presented at a Genethics UK Forum meeting. It relates to issues surrounding the sharing of genetic information within families, which is a frequently arising theme in Genethics UK Forum meetings. Genetic testing of one individual may reveal information that could also predict disease for their family members. This often raises questions about when and how such information might need to be imparted to relatives. The healthcare professional presenting the case described a serious heritable condition identified in a patient, who was then reluctant to communicate this to her adult children who might have inherited the condition. The healthcare professional had identified a high risk for the patient’s children. The question was therefore whether the health professional needed to alert the adult children to their risks. The details of the case have been changed to remove any potentially identifying patient details, and the health professional contributor has reflected on our modifications and our analysis of the case to check the meaning was not altered.

A middle-aged woman was referred to a specialist Clinical Genetics service as she was suspected of having a genetic condition. If this suspicion was confirmed through genetic testing, then her [adult] children would each have a 1 in 2 risk of having inherited the condition. The referral letter indicated that the patient did not want her children to know about their risks and so she was hesitant to undergo any genetic testing. This issue was explored with the patient who revealed that she had experienced a lot of anxiety and worry regarding her diagnosis and had not seen the benefits of preventative treatments. Despite the seriousness of the condition (a risk of premature death), the patient did not want to share the recommendation for screening with her children because she did not want them to experience the same level of anxiety and worry. However, screening could help identify early signs of the condition in the children, for which medical or surgical interventions could be offered to mitigate against the most serious consequences. The patient was adamant about her children not being contacted and became agitated when this was discussed. The health professional felt torn between respecting these wishes (and empathy for their cause) and the perceived obligation to alert the children about their risks.

In the below, we explore this case through the lens of COM-B.

### Capability (ability)

The healthcare professional was *capable* of recognising that the above case was morally complex and described the variety of conversations they had with the patient in an effort to understand the issue more fully: ‘*so we had a candid conversation* […] *especially around secret-keeping and how actually often that doesn’t work in families*.’ Such ‘*difficult conversation*[s]’ require skills and understanding to balance patient-clinician rapport, whilst bringing up contentious topics: ‘*I didn’t want her to go ‘off-side’, but it was also a conversation where I raised the possibility that her children could develop this condition and that actually to be safe* [in terms of surveillance for the children] *it was best that they were aware.’* Through the course of these discussions, the healthcare professional was able to empathise with both the patient and her relatives, whilst also keeping in mind professional guidelines and personal morals and values. Their skills and experience (*capability*) led them to recognise their ‘hunch’ that while the patient professed to think about the option of genetic testing after their meeting, ‘*it kind of left me feeling like, “actually, I don’t think she’s going to change her mind”’*, and that it would be useful to discuss the case with the Genethics community: ‘*I thought I’d just raise this with the group to see if there were any other ideas or if anyone else had any similar situations.*’ This meant the healthcare professional could evaluate, anticipate and plan for the ethical aspects between appointments.

As mentioned above, health professionals keep their knowledge and understanding (*capability*) of guidelines in mind as they carry out what may appear like conflicting duties. In this case study for example, the disclosure of genetic information with relevance to another individual is cited by the General Medical Council (GMC) as an example in which confidential medical information may be disclosed in cases where there is a ‘risk of death or serious harm.’ The healthcare professional is advised to ‘balance their duty to make the care of their patient their first concern’ against their ‘duty to help protect another person from serious harm,’ and where possible, not to disclose the identity of their patient [40]. Parker and Lucassen [41] propose a model to tease apart an individual’s personal health information (diagnosis of a disease), from the familial genetic information (the hereditary risk of disease); an approach which appears compatible with the GMC’s guidance to preserve individual confidentiality [42]. This position is further supported by the Joint Committee on Genomics in Medicine guidelines: Consent and Confidentiality in Genomic Medicine [43]. Guidelines facilitate the ethical decision-making process to an extent, offering guidance on general principles, however this is dependent on the health professional’s level of skill (experience, understanding and knowledge). Moreover, guidelines require interpretation in the context of details relevant to the case [44]. When a health professional’s understanding and knowledge of guidelines conflict with their own assessment (see motivation section), guidelines become less facilitatory and other mechanisms are needed to support the ethical decision-making process, such as discussion within the Genethics Forum as took place in this case.

### Opportunity

The healthcare professional had the *opportunity* to bring the case to their wider health professional team for discussion (‘*so I discussed it with the wider team*’), as well as to the wider community (Genethics). In both instances, the presence of this infrastructure represents both a *physical* and *social* opportunity; physical because the infrastructure was there, and social because those to whom the health professional spoke to were supportive (as illustrated by the interaction between the health professional and forum). The Genethics forum offers a space in which healthcare professionals can engage in moral practice by seeking out and exploring ethical issues, rather than offering a purely solution-based discussion [44].

#### ABC versus St George’s NHS trust

During discussions about the clinical case, several participants reflected on the outcome of a particular legal case that they thought might be useful to consider in this instance. Our analysis of this discussion suggested that there was a tendency for participants to lack confidence in their own ethical decision-making, preferring to turn to legal rules  even when working within the parameters of the law, despite the fact ethical decision-making might have been a more appropriate option. This perception appeared to be a key barrier to ethical preparedness and may be influenced by social opportunity. The legal case referred to was ABC vs St George’s NHS Trust case (ABC v St George's Healthcare NHS Trust [2020] EWHC 455 (QB)), which involved a patient who was diagnosed with Huntington’s disease but who did not give his consent for his pregnant daughter (ABC) to be told of his diagnosis.The case contributor described how other members of their department had suggested they seek legal advice because the ABC case was also about familial communication: ‘*lots of members of the team… felt maybe seeking some advice from our lawyers from the hospital would be useful in this case.’* Whilst some at the Genethics meeting thought such a referral was not necessary, another attendee thought that legal advice was now necessary because of the similarities with an established legal case. This attendee appeared to hold the view that the law would give a clear answer about what to do and that this was preferable (a safer option) to a professional judgement based on the contextual features of the case. Working within legal parameters is vital, but within these parameters being ethically prepared means realising that there is a certain amount of professional discretion that comes from the consideration of how to act ethically.

### Motivation (willingness)

The healthcare professional recognised the case as ethically contentious and sought wider professional input. Utilising their own experience and capability, they made the decision to discuss the case within the Genethics UK forum (reflective motivation) to capitalise on the experience and knowledge of the wider clinical community. It is possible that guidelines relevant to this area of medicine (described above) facilitate the motivation to be ethically prepared by compelling the health professional to think deeply about their particular case or issue. Though despite the professional guidelines regarding familial communication of genetic information, healthcare professionals still grapple with the practicalities of separating familial and personal genetic information [45, 46], and the fact that communicating genetic information with relatives against the wishes of a patient might disrupt family relationships and damage patient trust in health professionals [46]. This contrasts with the view held by many patients who see genetic information as familial, and support the sharing of genetic information in the absence of explicit consent where not to do so has potentially serious health consequences for at-risk relatives [45].

*Reflective motivation* was also demonstrated by the gathering of as much information about the patient’s motivation and context in order to better evaluate the ethical issue. The healthcare professional was encouraged by an attendee to involve other family members (not themselves at genetic risk) to support the patient and allow for another perspective to be heard. Another attendee also suggested thinking of other ways to gain more information by enquiring whether the patient had ever had general discussions regarding screening with her children, as a way for the patient to reflect on her children’s likely preferences.

### Summary

Overall, analysis of this case using the COM-B model, helps us understand the factors leading to ethical preparedness in this healthcare professional attending Genethics UK Forum. They are skilled (capable) at recognising issues, through their actions they show willingness (motivation) to reach a resolution, and they have the opportunity to discuss issues with colleagues in both local and national forums. We identified the main barrier to ethical preparedness in this case to be a misperception about the ability of the law to provide proscriptive answers to ethical issues and a lack of confidence in knowing when to apply professional judgement. To be more ethically prepared, healthcare professionals need to be able to make decisions about complex cases by incorporating ethical, legal and practical considerations; they need to understand how the law and ethics interact, and the most appropriate point at which referral to the law needs to take place.

## Discussion

While the concept of preparedness has typically been associated with public health emergencies, and where ethical preparedness has been used to indicate attention to governance systems, rapid technological advances in broader health research and care settings would benefit from attention to ethical preparedness. This is because these contexts typically involve the use of technologies that constantly push the boundaries of traditional ethical approaches to practice. We have articulated a concept of ethical preparedness that is applicable to both public health emergencies, as well as to these broader contexts. We have illustrated that the process of ethical decision-making needs to be thought of as a behaviour, that ethical preparedness is something that is enacted by an individual, group or organisation, and that ethical preparedness requires consideration of how ethics will be implemented into practice. We have used COM-B/TDF to analyse the elements of this implementation to anticipate and identify ethical issues and to take all reasonable steps to address those issues or decisions before or when they are encountered.

Applying this approach to ethical preparedness to our case studies highlighted that being ethically prepared needs to go beyond merely equipping health professionals with skills and knowledge, or providing research governance actors with ethical principles and frameworks. A focus on the physical and social opportunity to allow or support actors to utilise their skills and knowledge (or principles and frameworks), and a better understanding of how motivation plays an integral part, is also required.

From our approach we note two main challenges to being ethically prepared. First, even if barriers to being ethically prepared are identified, they may be difficult to remove. For example, physical opportunity (financial, material, resources, inanimate parts of the environmental system) cannot be changed at the individual level—these are system/organisational level changes and often changes that require investment. Furthermore, changing social norms or organisational cultures/motivations is also challenging [47]. For example, the term ‘social norm’ or ‘cultural norm’ is used in inconsistent ways in the behavioural sciences, making investigation difficult;  there are a range of socio-political and cultural factors that play a role in constructing environments within which social norms are developed; and  social norms are often internalised by individuals, creating social identities that impact on behaviour differently needing complex interventions to change. Second, ethical preparedness is not a solutionist approach: even if an individual (or group of individuals) are ethically prepared, ethical decision-making is still fraught with difficult and complex decisions with often no correct ‘answer’. Finally, even if an individual, group of individuals or organisation does have the capability, opportunity and motivation to make a particular ethically considered decision, this act of decision-making may cause changes to the macro layers of the system (e.g., there could be a backlash from stakeholders, key actors and/or the public about the decision), which will, in turn, have implications on the capability, opportunity and motivation of the decision [48]. This creates the need for an iterative process of behavioural and ethical analysis and highlights the importance of recognising the temporality of decision making, and the dynamic relationship between the decision-making and changes in the capability, opportunity and motivation over time.

The conceptualisation of ethical preparedness presented here was developed on four main assumptions that need to be made explicit. First, that preparedness itself is worthy of value—experience of the COVID-19 pandemic showed that even though governments were prepared for a pandemic, this had limited impact (see [49]); second, that preparedness can be viewed as a behaviour; third, that actors in our analyses actually *felt* ethically prepared (further empirical evidence would be required to explore this); and finally, that the systemisation and simplification of theories and behaviours into models such as COM-B are useful. Concerns have arisen that such models perpetuate a lack of variability in the field [50], for example, the COM-B/TDF has become so dominant in the field of health psychology that Ogden (2016) worries that ‘*dominant ideas become “black boxed” and accepted as truths as they move beyond debate or critique*’ (pg. 247). Though many scholars disagree (for example, see [51]).

## Conclusions

We have explored ethical preparedness as a behaviour, and through this, provided insight into the specific factors that are needed to promote ethical decision-making—both in the pandemic context as well as in areas of health research and medicine where there have been rapid recent technological developments. We hope this offers a useful starting point for further conceptual work around the notion of being ethically prepared.

## Data Availability

The datasets generated and/or analysed during the current study are not publicly available due to confidentiality reasons. The dataset is stored in a secure archive and available in anonymised form on reasonable request. Any interest in the data should be directed to the authors (e.g., Lisa Ballard: l.ballard@soton.ac.uk) who will consider the request in line with the consent agreements in place.
